# Supplementary material for: Impaired immune response drives age-dependent severity of COVID-19
Source: J Exp Med. Author manuscript; Available in PMC 2022 Dec 5. (PMC9499827; doi:10.1084/jem.20220621)
Supplement: Supplementary Material [file EMS154767-supplement-Supplementary_Material.pdf]

Figure S1 | Increased and prolonged replication of SARS-CoV-2 MA in mice lacking type I and/or type III IFN receptors.

A) Groups of adult mice (8-18-week-old) of the indicated genotypes were infected with 10^5 PFU SARS-CoV-2 MA. Upper airways were harvested at the indicated time points and viral load determined by plaque assay on Vero E6 cells. Data pooled from five independent experiments. Symbols represent individual mice (n=4-13 per group) and bars indicate mean ± SEM. Dashed line indicates detection limit. *P≤0.05, **P≤0.01, ***P≤0.001, ****P≤0.0001, one-way ANOVA with Tukey's multiple comparisons test.

B) Groups of 16-24 week-old *Ifnlr1*$^{-/-}$ (triangles) and *Ifnar1*$^{-/-}$ mice (squares) were intranasally treated with the indicated dose of IFN-$\alpha_{B/D}$ or IFN-λ1/3, respectively, or mock-treated one day prior to infection with 10^5 PFU SARS-CoV-2 MA. Lung viral loads on day 3 p. i. were determined by plaque assay on Vero E6 cells. Data from a single experiment are shown. Symbols represent individual mice (n=6-7 per group) and bars indicate mean ± SEM. Dashed line indicates detection limit. ****P≤0.0001, one-way ANOVA with Tukey's multiple comparisons test.

C-F) Groups of adult (8-10-week-old) WT or *Ifnar1*$^{-/-}$ *Ifnlr1*$^{-/-}$ mice were mock-treated or infected with 10^5 PFU SARS-CoV-2 MA (n=2-3 per group) and prepared for histological analyses by cardiac perfusion. Antigen and histopathologic lesion scores for lungs and necrotizing bronchitis were quantified as described in materials and methods section. Data pooled from two independent experiments are shown. Symbols represent individual mice and bars indicate mean ± SEM.

G) Survival graph corresponding to Figure 1D.

H-J) Groups of adult (8-10-week-old; n=4) or aged *Ifnar1*$^{-/-}$ mice (36-52-week-old; n=5) were infected with 10^5 PFU SARS-CoV-2 MA. Mice were prepared for histological analyses by cardiac perfusion on day three post infection. Antigen and histopathologic lesion scores for bronchial necrosis were quantified as described in materials and methods section. *P≤0.05, **P≤0.01, unpaired t test.

K) Groups of adult or aged mice (8-12-week- or 40-60-week-old; n=4-7) of the indicated genotypes were infected with 10^5 PFU SARS-CoV-2 MA. Upper airways were harvested at the indicated time points and viral loads determined by plaque assay on Vero E6 cells. Data pooled from four independent experiments are shown. Symbols represent individual mice and bars indicate mean ± SEM. Dashed line indicates detection limit. *P≤0.05, unpaired t test.

Figure S2 | Serial passaging of MA SARS-CoV-2 in IFN receptor-deficient C57BL/6 mice allows for rapid host adaptation.

A-B) Viral load on day 3 p. i. in upper airways (**A**) and lungs (**B**) for passaging series WT A, WT B, DKO A and DKO B determined by plaque assay on Vero E6 cells. Dashed line indicates detection limit.

C) Weight loss (left panel) and survival (right panel) of adult C57BL/6 WT mice (13-15-week-old; n=6 per group) infected with $5x10^3$ PFU of plaque-purified virus stocks DKO A PP and DKO B PP. Data from a single experiment are shown. Symbols represent individual mice and bars indicate mean ± SEM. Dashed line indicates experimental endpoint due to animal welfare. ***P*≤0.01, ****P*≤0.001, two-way ANOVA with Šídák's multiple comparisons test.

D) Variant frequency plot from next-generation sequencing results for the passaging series DKO B and the plaque-purified "DKO B PP" virus stock. Variant frequencies are shown in comparison to Wuhan-Hu-1 (NC_045512.2). Amino acid changes present in DKO B PP are indicated in bold, changes in comparison to SARS-CoV-2 MA are highlighted in red.

E) Table indicating similar (orange) or identical (green) amino acid changes present in SARS-CoV-2 MA10, SARS2-$N501_{MA30}$, B1-1-529 (Omicron), B.1.351 (Beta) and P.1 (Gamma) in comparison with amino acid changes present in SARS-CoV-2 MA20 (red). Reference sequence: Wuhan-Hu-1 (NC_045512.2). Amino acid changes highlighted in grey were already present in SARS-CoV-2 MA.

F) ACE2 dependent entry of SARS-CoV-2 MA20 was evaluated by infecting ACE2 expressing A549 cells with an MOI of 0.1 in comparison with control A549 cells. Virus replication 4 d p.i. was quantified by plaque assay on Vero E6 cells. Data from a single experiment are shown. Symbols represent technical replicates and bars indicate mean ± SEM. Dashed line indicates detection limit. ***P*≤0.01, unpaired t test.

G) Comparative growth curves of B.1 and SARS-CoV-2 MA20 on VeroE6 cells infected with an MOI of 0.001. Virus replication was quantified by plaque assay on Vero E6 cells. Data from a single experiment performed in duplicates are shown. Dashed line indicates detection limit. ***P*≤0.01, two-way ANOVA with Tukey's multiple comparisons test.

H) Comparative neutralization by plaque reduction neutralization test of SARS-CoV-2 MA20 and B.1.617.2 (Delta) using sera from vaccinated individuals. Symbols represent mean value for each individual determined in three independent assays. ****P*≤0.001, paired t test.

I) Adult C57BL/6 mice (10-week-old; n=5 per group) were infected with 10^3 PFU of SARS-CoV-2 MA20. Upper airways were harvested at the indicated time points and gene expression levels of *Il6*, *Tnf*, *Ifna4*, *Ifnb*, *Ifnl2/3*, *Mx1*, *Isg15* and *Stat1* were determined relative to *Ubc* by RT-qPCR. Symbols represent mean ± SD.

Figure S3 | Increased disease susceptibility of aged mice is associated with an impaired type I IFN response.

A) Groups of adult or aged C57BL/6 (10-week- or 40-week-old; n = 5 per group) were infected with 100 PFU SARS-CoV-2 MA20. Weight loss (left panel) and survival (right panel) were monitored for 14 days post infection. Data from a single experiment are shown. Symbols represent mean ± SEM. Dashed line indicates experimental endpoint due to animal welfare. Survival: $^{**}P \leq 0.01$, Log-rank (Mantel-Cox) test. Dataset for infected 10-week-old mice is also blotted in Figure 2G.

B) Groups of adult or aged *Ifnlr1*$^{-/-}$ mice (12-week- or 52-week-old; n = 5 per group) were mock-treated or intranasally inoculated with 40 µg of poly (I:C). Lungs were harvested at the indicated time points and gene expression levels of *Ifnb1*, *Ifnl2/3*, *Mx1* and *Isg15* determined relative to *Ubc* by RT-qPCR. Data from a single experiment are shown. Symbols represent individual mice and bars indicate mean ± SEM. $^{*}P \leq 0.05$, $^{***}P \leq 0.001$, $^{****}P \leq 0.0001$, two-way ANOVA with Šídák's multiple comparisons test.

Figure S4 | Prophylactic IFN-α and therapeutic IFN-λ or IFN-γ treatment reduces SARS-CoV-2 induced lethality in highly susceptible mice.

A) Groups of adult *Ifnlr1*$^{-/-}$ mice (13-15-week-old) were either mock-treated (n=6) or treated prophylactically by subcutaneous injection of 2 µg IFN-$\alpha_{B/D}$ once one day prior to infection (n=7) with 1000 PFU SARS-CoV-2 MA20. Dashed line indicates experimental endpoint due to animal welfare. Data from a single experiment are shown. Symbols represent mean ± SEM. Weight loss: $^{****}P \leq 0.0001$, by two-way ANOVA with Šídák's multiple comparisons test. Survival: Log-rank (Mantel-Cox) test; $^{*}P \leq 0.05$.

B) Groups of aged WT mice (45-56-week-old) were either mock-treated (n=8) or treated therapeutically with 3 µg IFN-λ1/3 (n=8) daily for one week starting one day after infection with 200 PFU SARS-CoV-2 MA20. Mock-treated control group is the same as depicted in Figure 7C. Data from a single experiment are shown. Dashed line indicates experimental endpoint due to animal welfare. Symbols represent mean ± SEM. Weight loss: $P \leq 0.05$ by two-way ANOVA with Šídák's multiple comparisons test. Survival: Log-rank (Mantel-Cox) test, $^{*}P \leq 0.05$.

C) Groups of aged WT mice (48-52-week-old; n=7) were treated and infected as depicted in (**B**). Organs were harvested on day 4 p. i.. Viral replication in upper airways was quantified as SARS-CoV-2 genome equivalents per ml by measuring expression levels of the viral gene *E* by RT-qPCR Lung (left panel) and viral loads in lungs were determined by plaque assay on Vero E6 cells (right panel. Data from a single experiment are shown. Symbols represent individual mice and bars indicate mean ± SEM. $^{****}P \leq 0.001$, unpaired t test.

D) Groups of aged *Ifnar1*$^{-/-}$ mice (52-60-week-old) were either mock-treated (n=9) or treated therapeutically by subcutaneous injection of 3 µg IFN-γ daily for one week (n=8) starting one day after infection with 100 PFU SARS-CoV-2 MA20. Mock-treated control group is the same as depicted in Figure 7E. Data from a single experiment are shown. Dashed line indicates experimental endpoint due to animal welfare. Survival: Log-rank (Mantel-Cox) test, $^{*}P \leq 0.05$.

Figure S5 | Impaired immune response drives age-dependent virulence of SARS-CoV-2

Graphical summary illustrating the age-dependent impairment of immune responses and suggested intervention strategies.

Supplementary Table S1: Histological scoring criteria

Criteria no.	Criteria	Scoring description*
1	atelectasis (pneumonia-associated), area affected	0 = no change, 1 = focal to oligofocal <5%, 2 = multifocal (6-40%), 3 = coalescing (41-80%), 4 = diffuse >80%
2	infiltrates alveolar, area affected	0 = no change, 1 = focal to oligofocal <5%, 2 = multifocal (6-40%), 3 = coalescing (41-80%), 4 = diffuse >80%
3	infiltrates alveolar, grade	0 = no infiltrates, 1 = 1-2 cells, 2 = 2-3 cells, 3 = almost filling, 4 = full atelektasis, grade is representative for the lobe
	description: predominant cell type	granulocytes, lymphocytes, macrophages, plasma cells, mixture (no predominant cell type)
4	infiltrates interstitial, area affected	0 = no change, 1 = focal to oligofocal <5%, 2 = multifocal (6-40%), 3 = coalescing (41-80%), 4 = diffuse >80%
5	infiltrates interstitial, grade	0 = no infiltrates, 1 = subtle, 2 = thickend septa but regular alveolar pattern, 3 = thickened septa partially obscured alveolar architecture, 4 = full atelektasis; grade is representative for lobe
	description: predominant cell type	granulocytes, lymphocytes, macrophages, plasma cells, mixture (no predominant cell type)
6	infiltrates peribronchial (incl glands), occurence	0 = not present, 1 = present in 1-3 foci, 3 = significantly present in >3 foci
7	infiltrates peribronchial, grade	= cell layers; 1=1, 2=2-3, 3=4-5, 4≥6 give max grade
	description: predominant cell type	granulocytes, lymphocytes, macrophages, plasma cells, mixture (no predominant cell type)
8	necrotizing bronchitis, occurence	0 = not present, 1 = present in 1-3 foci, 3 = significantly present in >3 foci
9	necrotizing bronchitis, grade	for infiltrates /debris(1=few cells, 2 aggregates, 3 almost filling; 4 > filling lumen), grade max
	description: predominant cell type	granulocytes, lymphocytes, macrophages, plasma cells, mixture (no predominant cell type)
10	mucus increased	main = 1; periphery = 2
11	infiltrates perivascular, occurence	0 = not present, 1 = present in 1-3 foci, 3 = significantly present in >3 foci
12	infiltrates perivascular, grade	0 = no infiltrates, 1 = 1 cell layer, 2 = 2-3 layers, 3 = 4-5 layers, 4 = ≥6 layers, maximum score given
	description: predominant cell type	granulocytes, lymphocytes, macrophages, plasma cells, mixture (no predominant cell type)
13	vascular rolling of immune cell, occurence	0 = not present, 1 = present in 1-3 foci, 3 = significantly present in >3 foci
14	endotheliitis, occurence	0 = not present, 1 = present in 1-3 foci, 3 = significantly present in >3 foci
15	vasculitis, i.e. mural damage, transmigrating immune cells	0 = not present, 1 = present in 1-3 foci, 3 = significantly present in >3 foci
16	thrombi, occurence	0 = not present, 1 = present
17	necrosis, alveolar epithelium, area affected	0 = no change, 1 = focal to oligofocal <5%, 2 = multifocal (6-40%), 3 = coalescing (41-80%), 4 = diffuse >80%
18	diffuse alveolar damage, with hyaline membrane, debris, fibrin, area affected	0 = no change, 1 = focal to oligofocal <5%, 2 = multifocal (6-40%), 3 = coalescing (41-80%), 4 = diffuse >80%
19	hypertrophy/hyperplasia, bronchi, occurence	0 = not present, 1 = present in 1-3 foci, 3 = significantly present in >3 foci
20	hyperplasia /hypertrophy type II pneumocytes, area affected	0 = no change, 1 = focal to oligofocal <5%, 2 = multifocal (6-40%), 3 = coalescing (41-80%), 4 = diffuse >80%
21	atypical cells or syncytia, occurence	0 = not present, 1 = present
**sum scores**	**bronchial necrosis**	**sum of no. 8 to 9**
	**necrotizing bronchitis**	**sum of no. 6 to 9**
	**regeneration**	**sum of no. 19 to 20**
	**total lung score**	**sum of no. 1 to 21**

*scores can be translated to minimal (score 1), mild (score 2), moderate (score 3), severe (score 4)

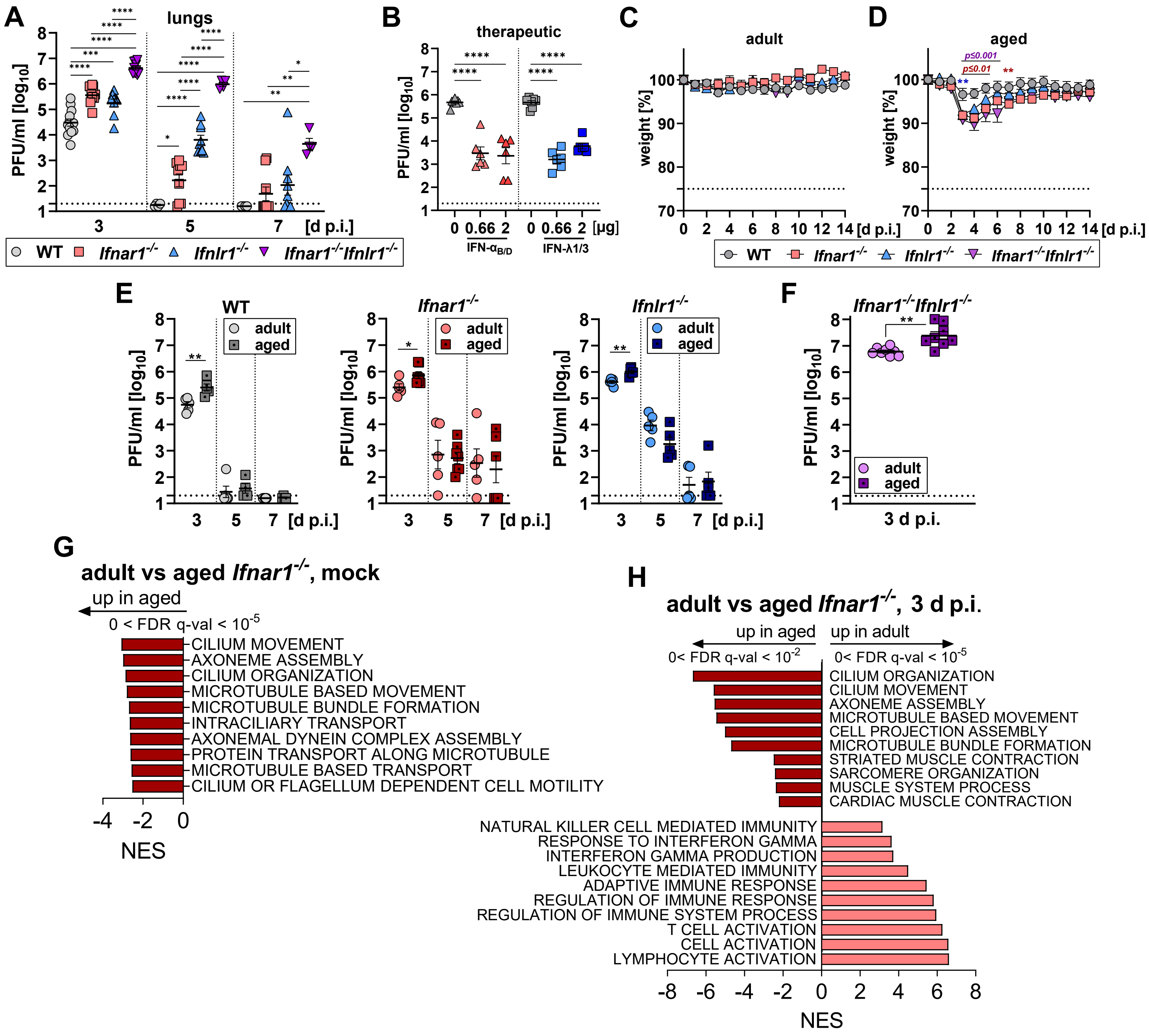
A
lungs
PFU/ml [log10]
3
5
7
[d p.i.]
WT
Ifnar1-/-
Ifnlr1-/-
Ifnar1-/-Ifnlr1-/-
B
therapeutic
PFU/ml [log10]
0 0.66 2 0 0.66 2 [µg]
IFN-αB/D
IFN-λ1/3
C
adult
weight [%]
[d p.i.]
D
aged
weight [%]
p≤0.001
p≤0.01
[d p.i.]
WT
Ifnar1-/-
Ifnlr1-/-
Ifnar1-/-Ifnlr1-/-
E
WT
adult
aged
PFU/ml [log10]
[d p.i.]
Ifnar1-/-
Ifnlr1-/-
F
Ifnar1-/-Ifnlr1-/-
3 d p.i.
G
adult vs aged Ifnar1-/-, mock
up in aged
0 < FDR q-val < 10-5
CILIUM MOVEMENT
AXONEME ASSEMBLY
CILIUM ORGANIZATION
MICROTUBULE BASED MOVEMENT
MICROTUBULE BUNDLE FORMATION
INTRACILIARY TRANSPORT
AXONEMAL DYNEIN COMPLEX ASSEMBLY
PROTEIN TRANSPORT ALONG MICROTUBULE
MICROTUBULE BASED TRANSPORT
CILIUM OR FLAGELLUM DEPENDENT CELL MOTILITY
NES
H
adult vs aged Ifnar1-/-, 3 d p.i.
up in aged
up in adult
0< FDR q-val < 10-2
0< FDR q-val < 10-5
CILIUM ORGANIZATION
CILIUM MOVEMENT
AXONEME ASSEMBLY
MICROTUBULE BASED MOVEMENT
CELL PROJECTION ASSEMBLY
MICROTUBULE BUNDLE FORMATION
STRIATED MUSCLE CONTRACTION
SARCOMERE ORGANIZATION
MUSCLE SYSTEM PROCESS
CARDIAC MUSCLE CONTRACTION
NATURAL KILLER CELL MEDIATED IMMUNITY
RESPONSE TO INTERFERON GAMMA
INTERFERON GAMMA PRODUCTION
LEUKOCYTE MEDIATED IMMUNITY
ADAPTIVE IMMUNE RESPONSE
REGULATION OF IMMUNE RESPONSE
REGULATION OF IMMUNE SYSTEM PROCESS
T CELL ACTIVATION
CELL ACTIVATION
LYMPHOCYTE ACTIVATION
NES

Figure 1

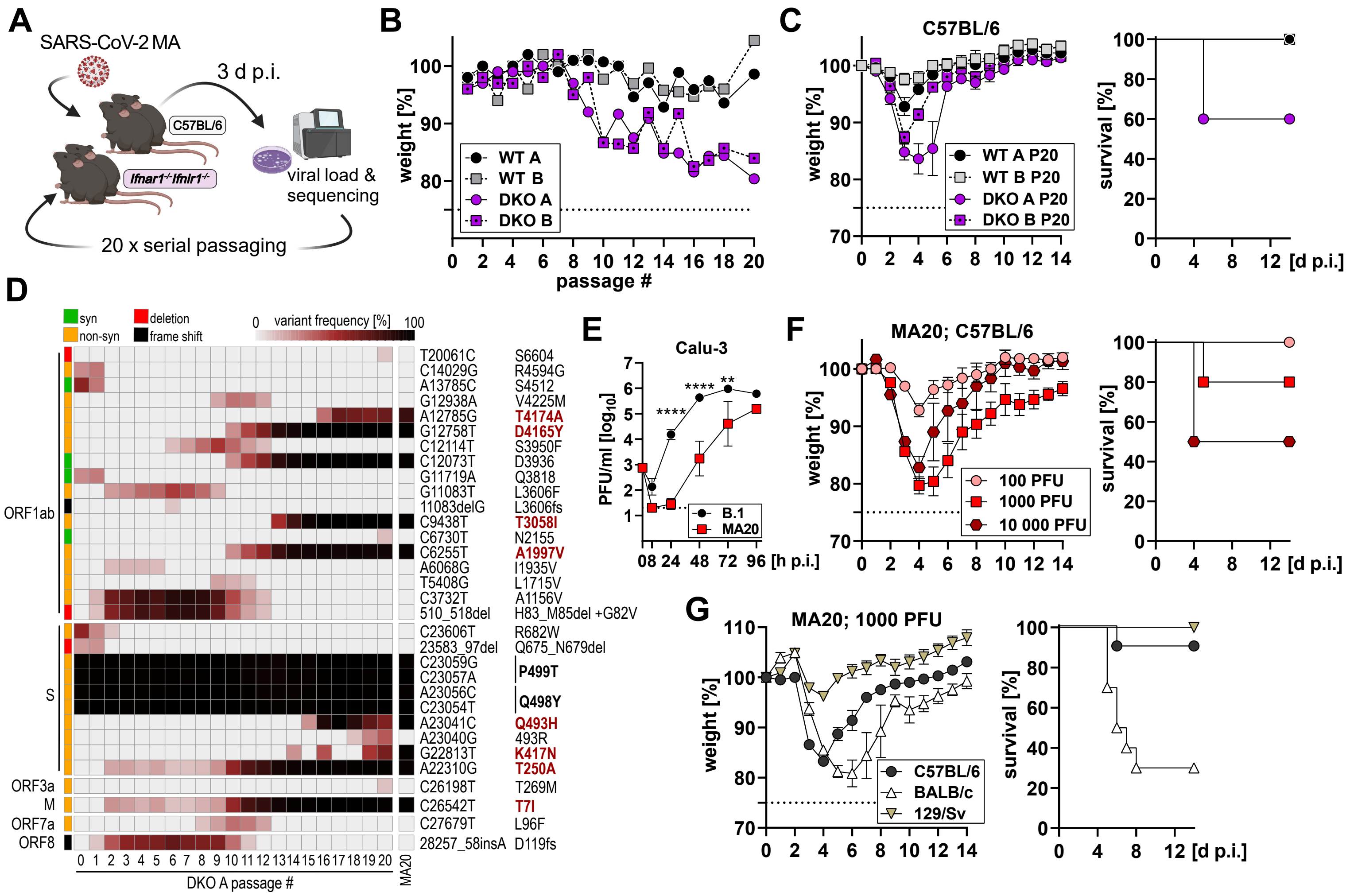
A
SARS-CoV-2 MA
3 d p.i.
C57BL/6
Ifnar1-/-Ifnlr1-/-
viral load & sequencing
20 x serial passaging
B
weight [%]
passage #
WT A
WT B
DKO A
DKO B
C
C57BL/6
WT A P20
WT B P20
DKO A P20
DKO B P20
survival [%]
[d p.i.]
D
syn
non-syn
deletion
frame shift
variant frequency [%]
ORF1ab
S
ORF3a
M
ORF7a
ORF8
T20061C S6604
C14029G R4594G
A13785C S4512
G12938A V4225M
A12785G T4174A
G12758T D4165Y
C12114T S3950F
C12073T D3936
G11719A Q3818
G11083T L3606F
11083delG L3606fs
C9438T T3058I
C6730T N2155
C6255T A1997V
A6068G I1935V
T5408G L1715V
C3732T A1156V
510_518del H83_M85del +G82V
C23606T R682W
23583_97del Q675_N679del
C23059G
C23057A P499T
A23056C
C23054T Q498Y
A23041C Q493H
A23040G 493R
G22813T K417N
A22310G T250A
C26198T T269M
C26542T T7I
C27679T L96F
28257_58insA D119fs
DKO A passage #
MA20
E
Calu-3
PFU/ml [log10]
B.1
MA20
[h p.i.]
F
MA20; C57BL/6
100 PFU
1000 PFU
10 000 PFU
G
MA20; 1000 PFU
C57BL/6
BALB/c
129/Sv

Figure 2

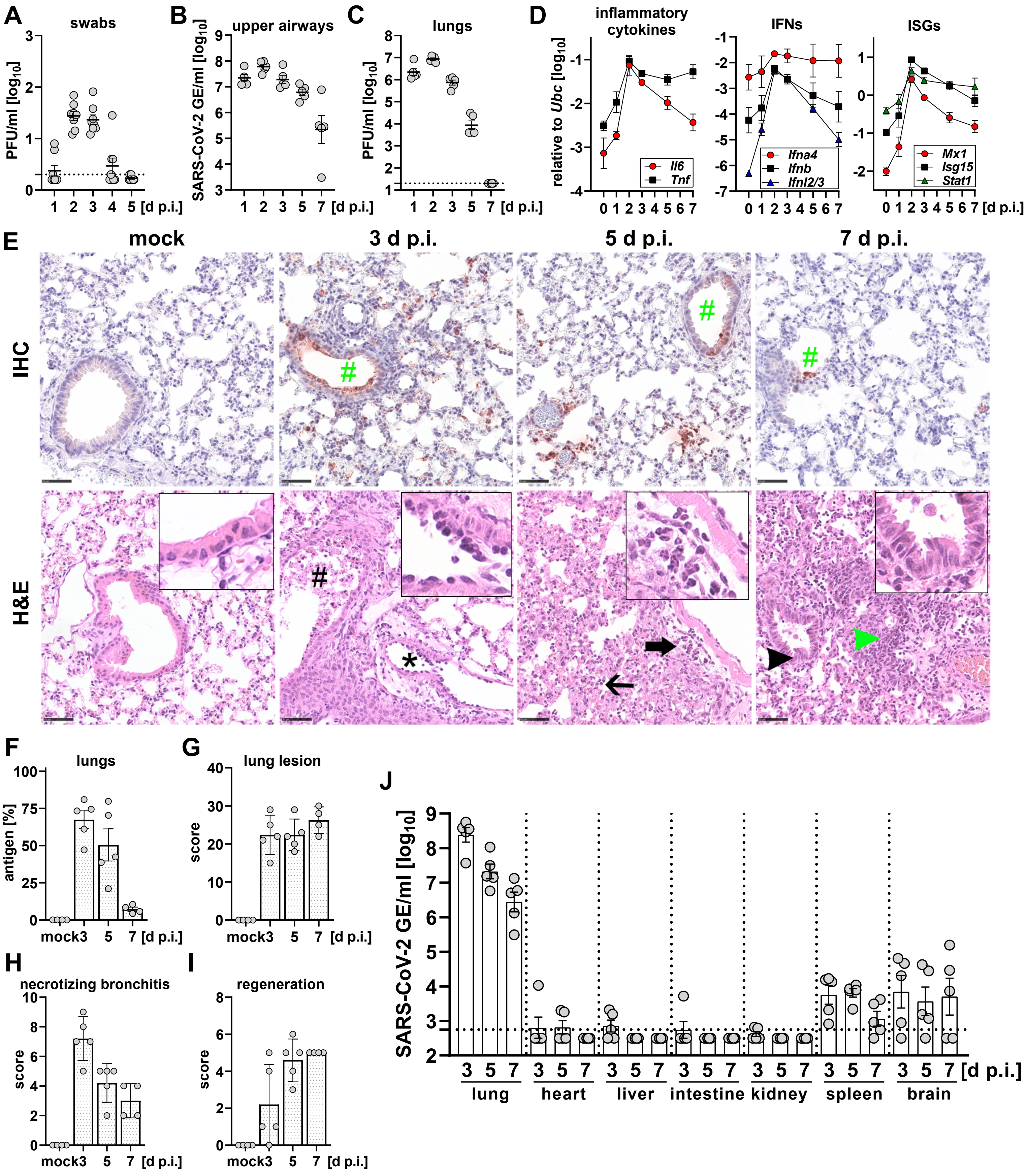

A
swabs
PFU/ml [log10]
1 2 3 4 5 [d p.i.]
B
upper airways
SARS-CoV-2 GE/ml [log10]
1 2 3 5 7 [d p.i.]
C
lungs
PFU/ml [log10]
1 2 3 5 7 [d p.i.]
D
inflammatory cytokines
IFNs
ISGs
relative to Ubc [log10]
Il6
Tnf
Ifna4
Ifnb
Ifnl2/3
Mx1
Isg15
Stat1
0 1 2 3 4 5 6 7 [d p.i.]
E
mock
3 d p.i.
5 d p.i.
7 d p.i.
IHC
H&E
F
lungs
antigen [%]
mock 3 5 7 [d p.i.]
G
lung lesion
score
mock 3 5 7 [d p.i.]
H
necrotizing bronchitis
score
mock 3 5 7 [d p.i.]
I
regeneration
score
mock 3 5 7 [d p.i.]
J
SARS-CoV-2 GE/ml [log10]
3 5 7 [d p.i.]
lung
heart
liver
intestine
kidney
spleen
brain

Figure 3

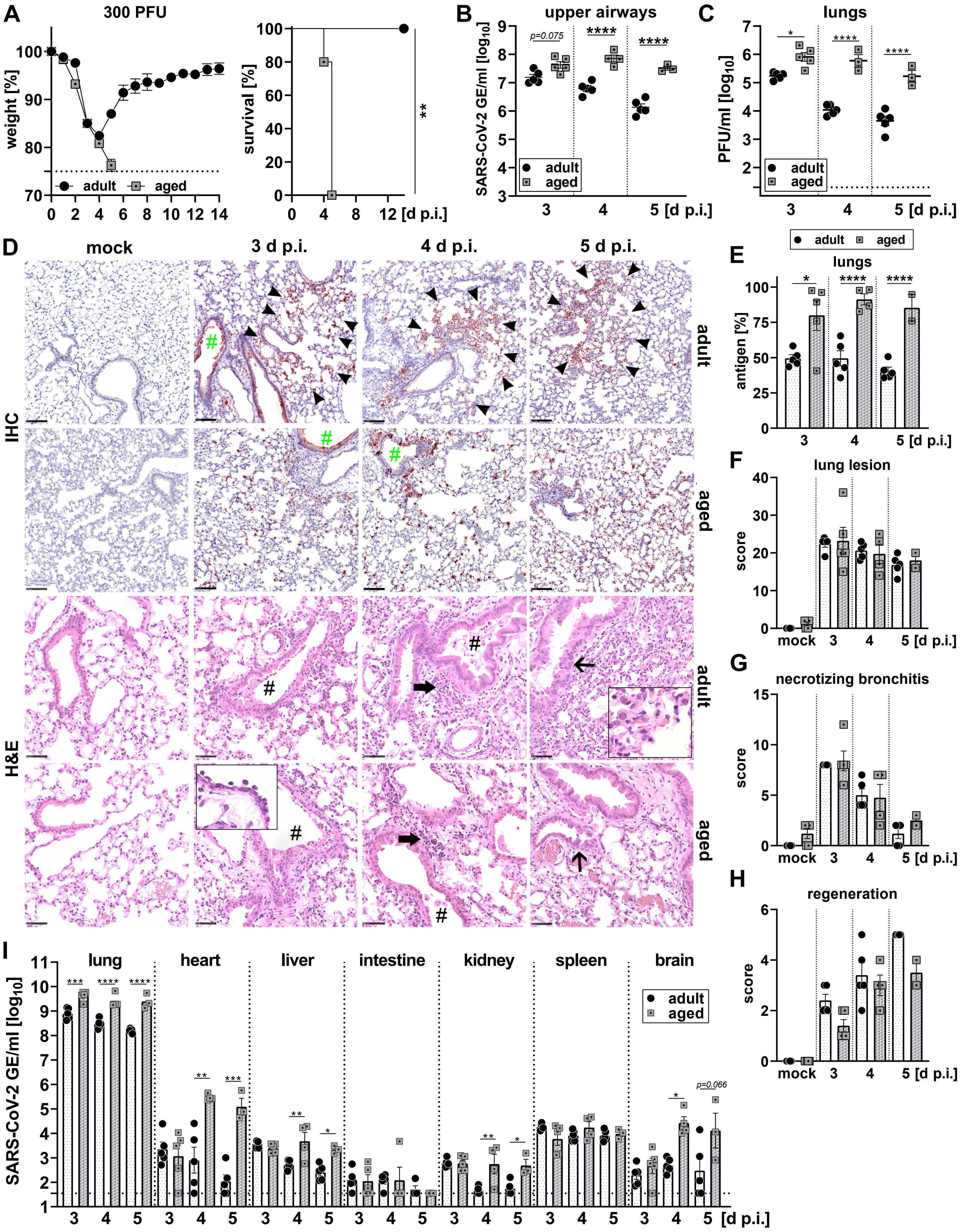
A
300 PFU
weight [%]
adult
aged
survival [%]
12 [d p.i.]
**
B
upper airways
SARS-CoV-2 GE/ml [log10]
p=0.075
****
5 [d p.i.]
C
lungs
PFU/ml [log10]
*
D
mock
3 d p.i.
4 d p.i.
5 d p.i.
IHC
H&E
adult
aged
E
antigen [%]
F
lung lesion
score
G
necrotizing bronchitis
H
regeneration
I
lung
heart
liver
intestine
kidney
spleen
brain
p=0.066
[d p.i.]

Figure 4

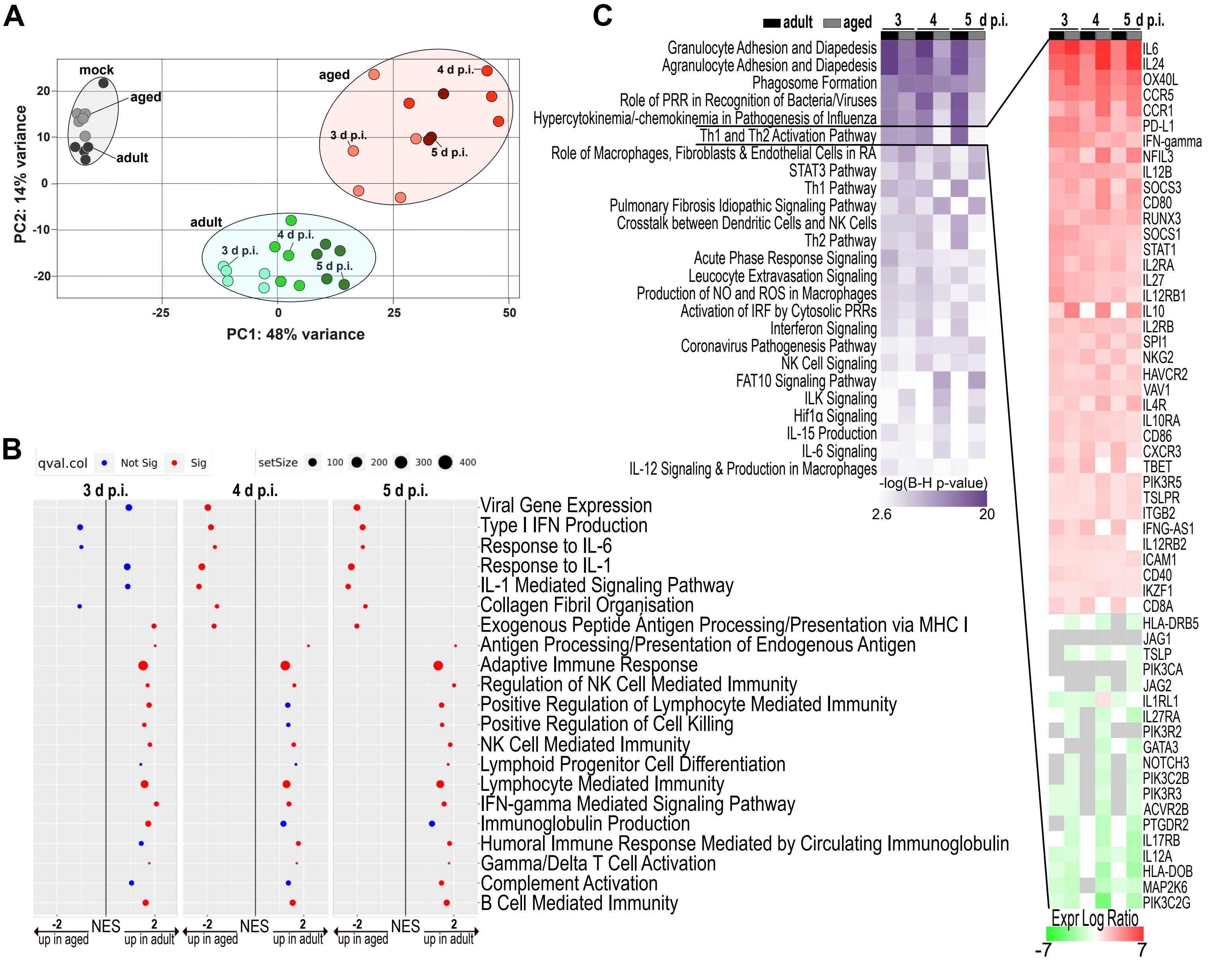
A
mock
aged
adult
aged
4 d p.i.
3 d p.i.
5 d p.i.
adult
4 d p.i.
3 d p.i.
5 d p.i.
PC2: 14% variance
PC1: 48% variance
B
qval.col Not Sig Sig
setSize 100 200 300 400
3 d p.i.
4 d p.i.
5 d p.i.
Viral Gene Expression
Type I IFN Production
Response to IL-6
Response to IL-1
IL-1 Mediated Signaling Pathway
Collagen Fibril Organisation
Exogenous Peptide Antigen Processing/Presentation via MHC I
Antigen Processing/Presentation of Endogenous Antigen
Adaptive Immune Response
Regulation of NK Cell Mediated Immunity
Positive Regulation of Lymphocyte Mediated Immunity
Positive Regulation of Cell Killing
NK Cell Mediated Immunity
Lymphoid Progenitor Cell Differentiation
Lymphocyte Mediated Immunity
IFN-gamma Mediated Signaling Pathway
Immunoglobulin Production
Humoral Immune Response Mediated by Circulating Immunoglobulin
Gamma/Delta T Cell Activation
Complement Activation
B Cell Mediated Immunity
NES
up in aged
up in adult
C
adult
aged
3 4 5 d p.i.
Granulocyte Adhesion and Diapedesis
Agranulocyte Adhesion and Diapedesis
Phagosome Formation
Role of PRR in Recognition of Bacteria/Viruses
Hypercytokinemia/-chemokinemia in Pathogenesis of Influenza
Th1 and Th2 Activation Pathway
Role of Macrophages, Fibroblasts & Endothelial Cells in RA
STAT3 Pathway
Th1 Pathway
Pulmonary Fibrosis Idiopathic Signaling Pathway
Crosstalk between Dendritic Cells and NK Cells
Th2 Pathway
Acute Phase Response Signaling
Leucocyte Extravasation Signaling
Production of NO and ROS in Macrophages
Activation of IRF by Cytosolic PRRs
Interferon Signaling
Coronavirus Pathogenesis Pathway
NK Cell Signaling
FAT10 Signaling Pathway
ILK Signaling
Hif1α Signaling
IL-15 Production
IL-6 Signaling
IL-12 Signaling & Production in Macrophages
-log(B-H p-value)
2.6
20
IL6
IL24
OX40L
CCR5
CCR1
PD-L1
IFN-gamma
NFIL3
IL12B
SOCS3
CD80
RUNX3
SOCS1
STAT1
IL2RA
IL27
IL12RB1
IL10
IL2RB
SPI1
NKG2
HAVCR2
VAV1
IL4R
IL10RA
CD86
CXCR3
TBET
PIK3R5
TSLPR
ITGB2
IFNG-AS1
IL12RB2
ICAM1
CD40
IKZF1
CD8A
HLA-DRB5
JAG1
TSLP
PIK3CA
JAG2
IL1RL1
IL27RA
PIK3R2
GATA3
NOTCH3
PIK3C2B
PIK3R3
ACVR2B
PTGDR2
IL17RB
IL12A
HLA-DOB
MAP2K6
PIK3C2G
Expr Log Ratio
-7
7

Figure 5

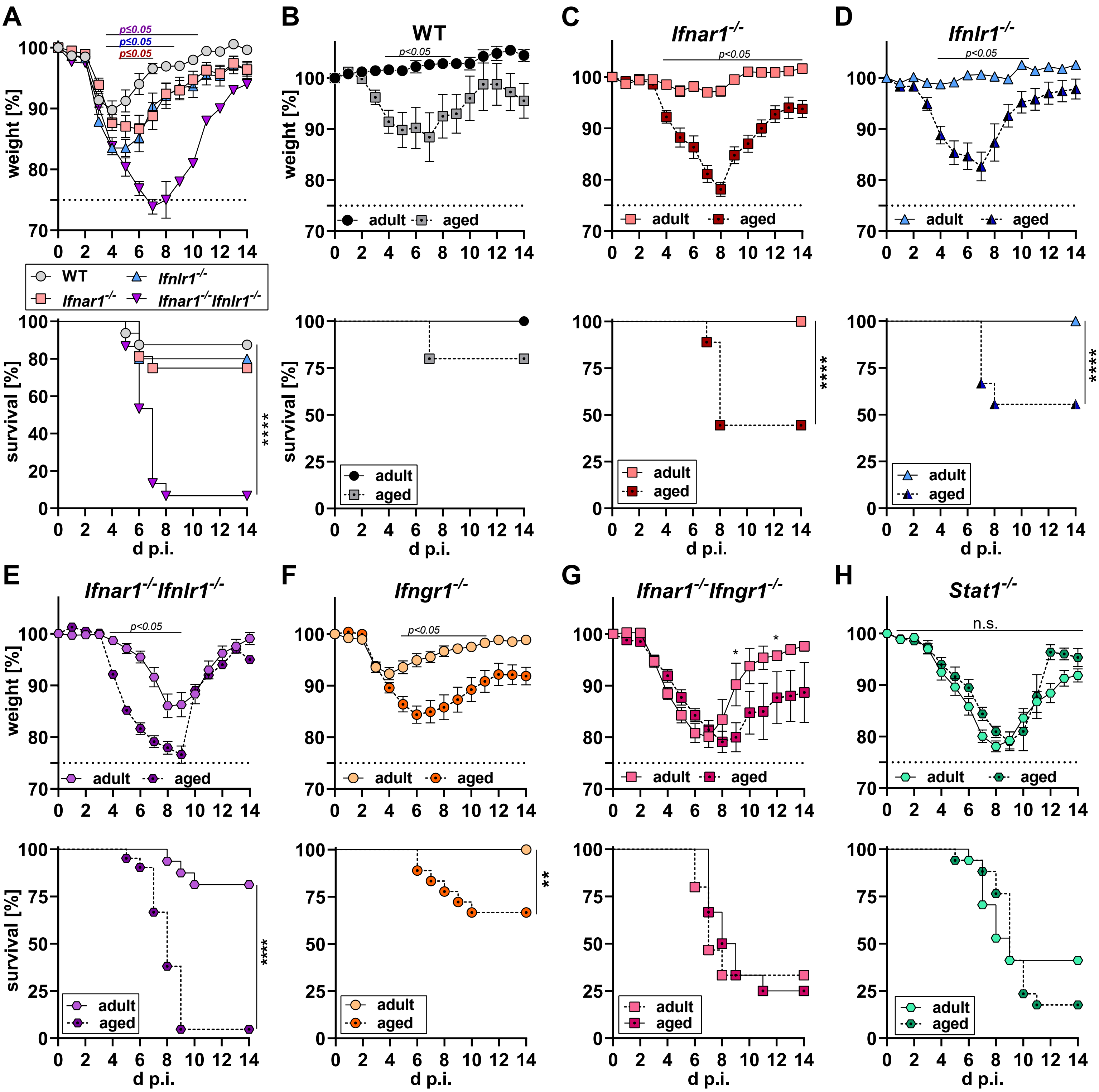
A
p≤0.05
p≤0.05
p≤0.05
weight [%]
WT
Ifnar1-/-
Ifnlr1-/-
Ifnar1-/-Ifnlr1-/-
survival [%]
****
d p.i.
B
WT
p<0.05
adult
aged
C
Ifnar1-/-
p<0.05
****
D
Ifnlr1-/-
p<0.05
****
E
Ifnar1-/-Ifnlr1-/-
p<0.05
****
F
Ifngr1-/-
p<0.05
**
G
Ifnar1-/-Ifngr1-/-
*
*
H
Stat1-/-
n.s.

Figure 6

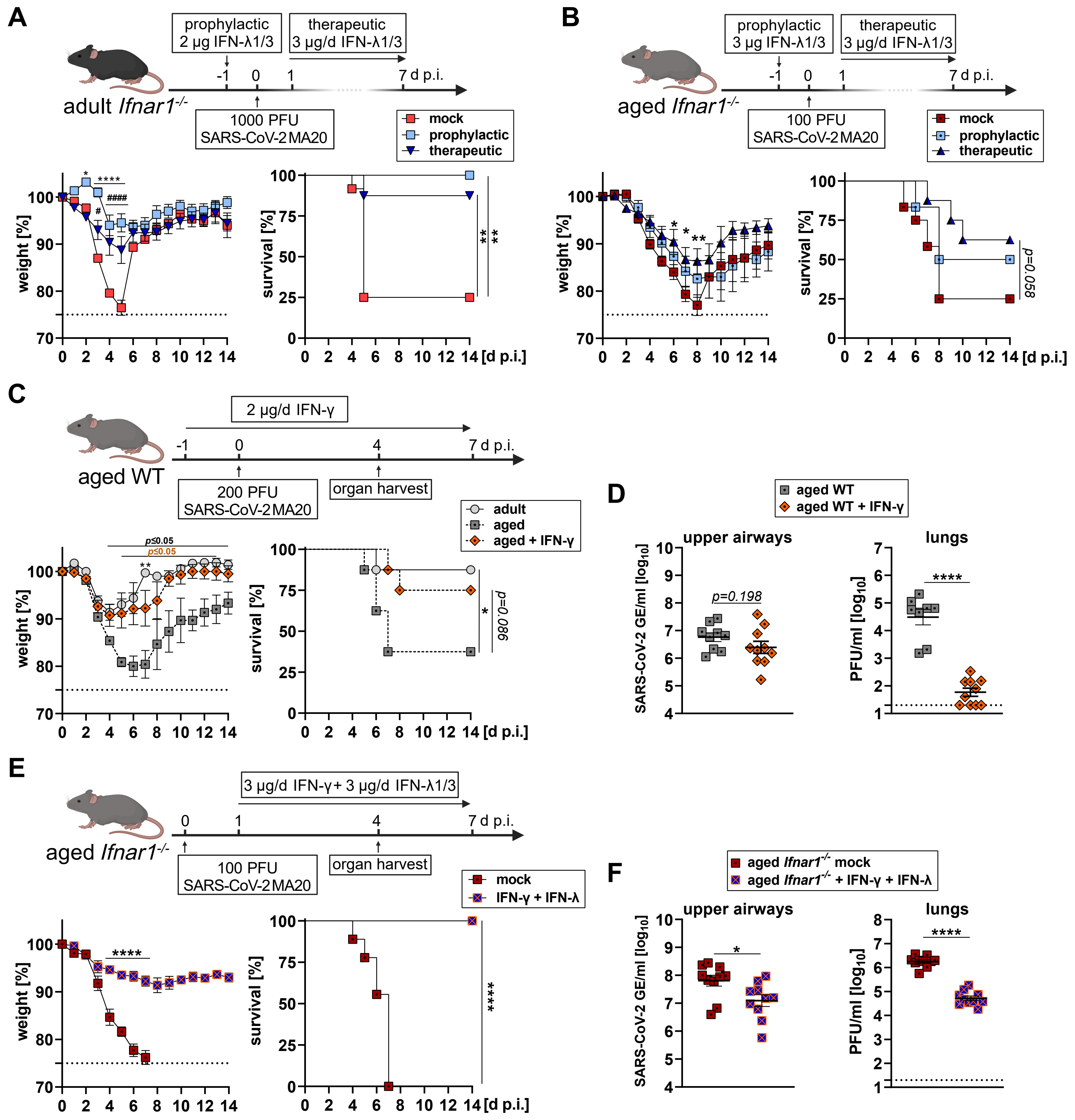
A
prophylactic
2 µg IFN-λ1/3
therapeutic
3 µg/d IFN-λ1/3
-1 0 1 7 d p.i.
adult Ifnar1-/-
1000 PFU
SARS-CoV-2 MA20
mock
prophylactic
therapeutic
weight [%]
survival [%]
[d p.i.]
B
prophylactic
3 µg IFN-λ1/3
therapeutic
3 µg/d IFN-λ1/3
aged Ifnar1-/-
100 PFU
SARS-CoV-2 MA20
p=0.058
C
2 µg/d IFN-γ
aged WT
200 PFU
SARS-CoV-2 MA20
organ harvest
adult
aged
aged + IFN-γ
p≤0.05
p=0.086
D
aged WT
aged WT + IFN-γ
upper airways
lungs
SARS-CoV-2 GE/ml [log10]
PFU/ml [log10]
p=0.198
E
3 µg/d IFN-γ + 3 µg/d IFN-λ1/3
aged Ifnar1-/-
100 PFU
SARS-CoV-2 MA20
organ harvest
mock
IFN-γ + IFN-λ
F
aged Ifnar1-/- mock
aged Ifnar1-/- + IFN-γ + IFN-λ
upper airways
lungs
SARS-CoV-2 GE/ml [log10]
PFU/ml [log10]

Figure 7

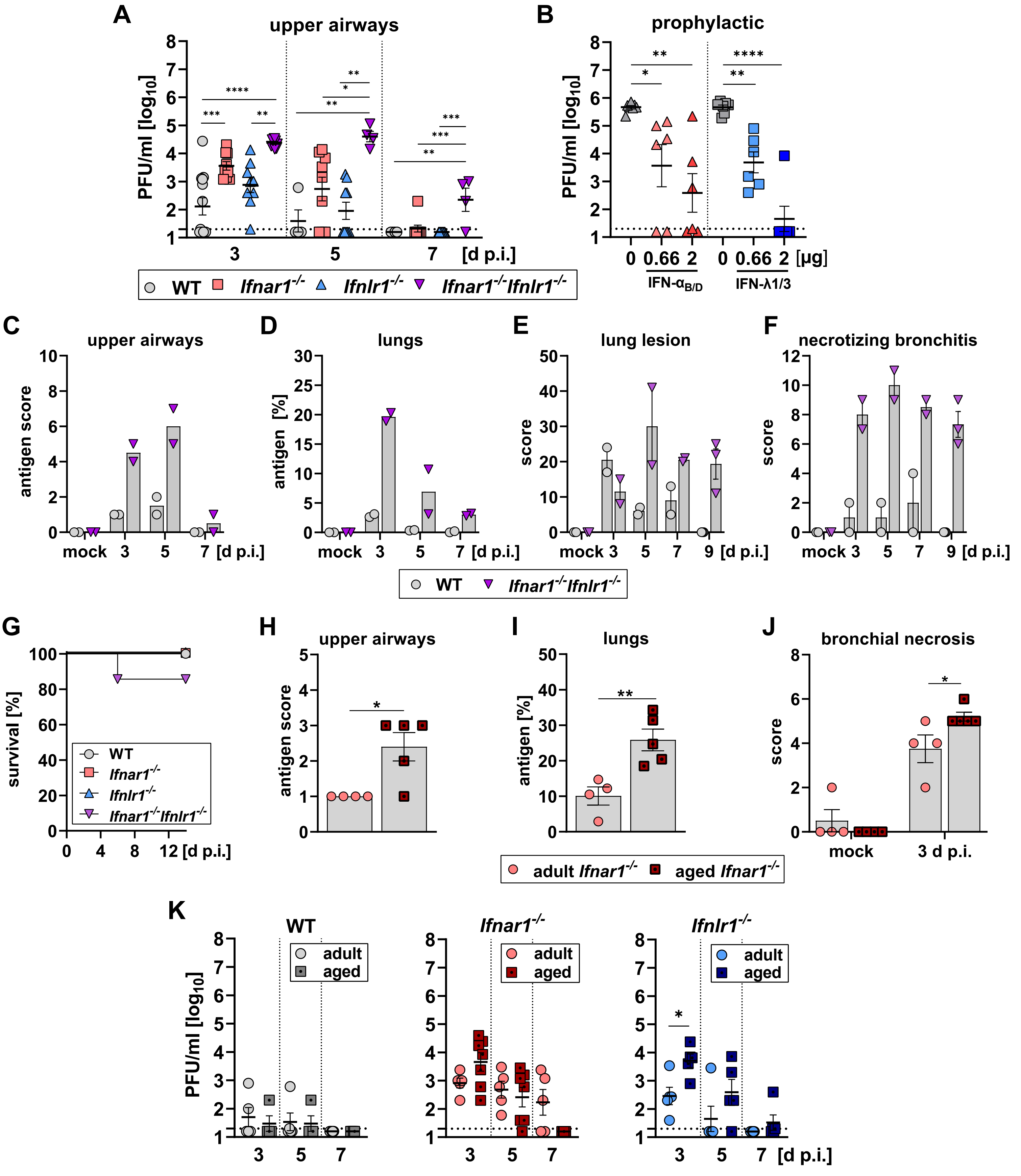

A
upper airways
PFU/ml [log10]
3
5
7
[d p.i.]
WT
Ifnar1-/-
Ifnlr1-/-
Ifnar1-/-Ifnlr1-/-
B
prophylactic
0
0.66
2
[µg]
IFN-αB/D
IFN-λ1/3
C
upper airways
antigen score
D
lungs
antigen [%]
E
lung lesion
score
F
necrotizing bronchitis
mock
9 [d p.i.]
G
survival [%]
12 [d p.i.]
H
upper airways
I
lungs
J
bronchial necrosis
3 d p.i.
adult Ifnar1-/-
aged Ifnar1-/-
K
adult
aged

Figure S1

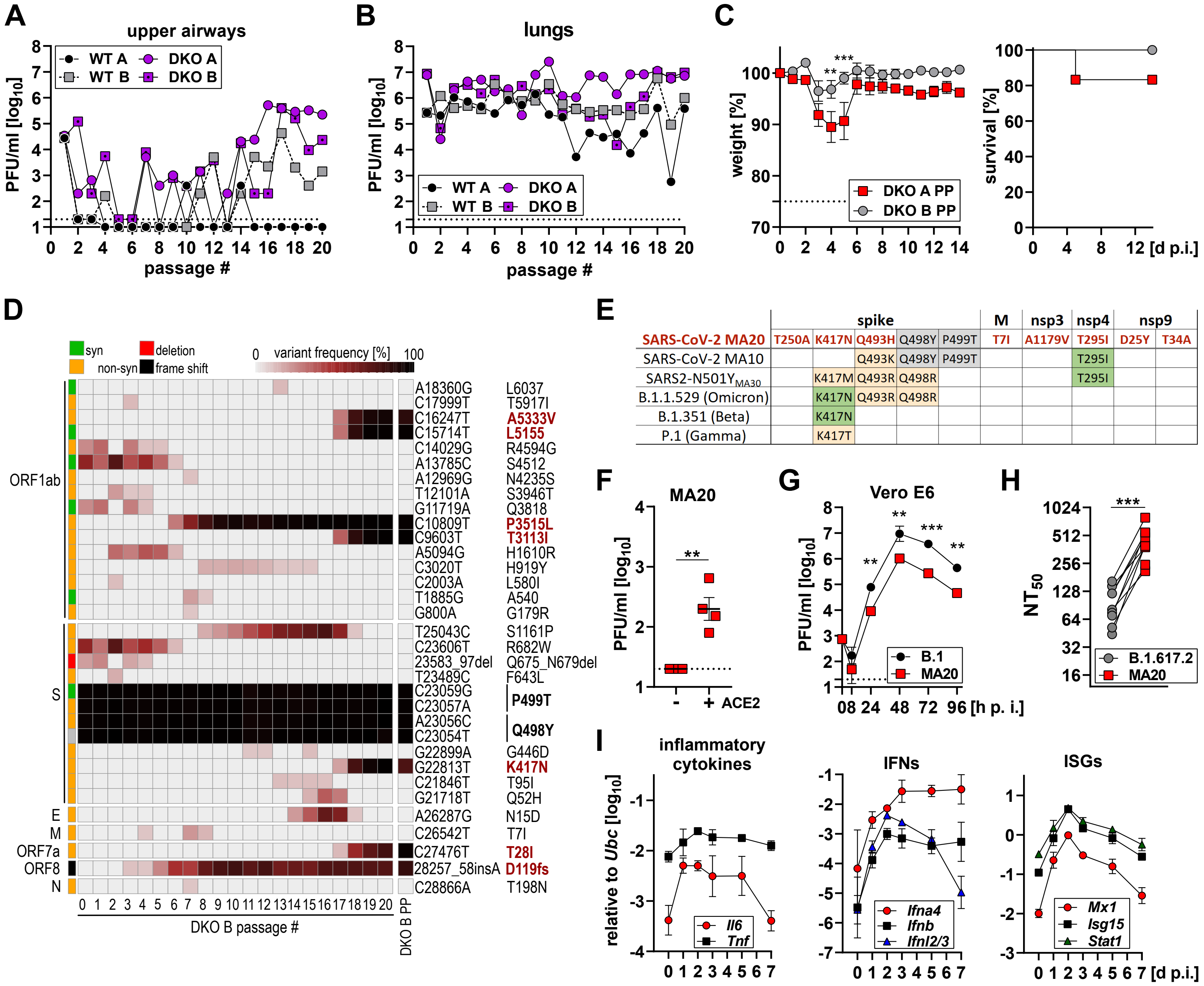

A
upper airways
WT A
DKO A
WT B
DKO B
PFU/ml [log10]
passage #
B
lungs
C
weight [%]
DKO A PP
DKO B PP
survival [%]
[d p.i.]
D
syn
non-syn
deletion
frame shift
variant frequency [%]
ORF1ab
S
E
M
ORF7a
ORF8
N
A18360G L6037
C17999T T5917I
C16247T A5333V
C15714T L5155
C14029G R4594G
A13785C S4512
A12969G N4235S
T12101A S3946T
G11719A Q3818
C10809T P3515L
C9603T T3113I
A5094G H1610R
C3020T H919Y
C2003A L580I
T1885G A540
G800A G179R
T25043C S1161P
C23606T R682W
23583_97del Q675_N679del
T23489C F643L
C23059G
C23057A
P499T
A23056C
C23054T
Q498Y
G22899A G446D
G22813T K417N
C21846T T95I
G21718T Q52H
A26287G N15D
C26542T T7I
C27476T T28I
28257_58insA D119fs
C28866A T198N
DKO B passage #
DKO B PP
E
spike
M
nsp3
nsp4
nsp9
SARS-CoV-2 MA20
T250A
K417N
Q493H
Q498Y
P499T
T7I
A1179V
T295I
D25Y
T34A
SARS-CoV-2 MA10
Q493K
Q498Y
P499T
T295I
SARS2-N501YMA30
K417M
Q493R
Q498R
T295I
B.1.1.529 (Omicron)
K417N
Q493R
Q498R
B.1.351 (Beta)
K417N
P.1 (Gamma)
K417T
F
MA20
ACE2
G
Vero E6
B.1
MA20
[h p. i.]
H
NT50
B.1.617.2
MA20
I
inflammatory cytokines
relative to Ubc [log10]
Il6
Tnf
IFNs
Ifna4
Ifnb
Ifnl2/3
ISGs
Mx1
Isg15
Stat1
[d p.i.]

Figure S2

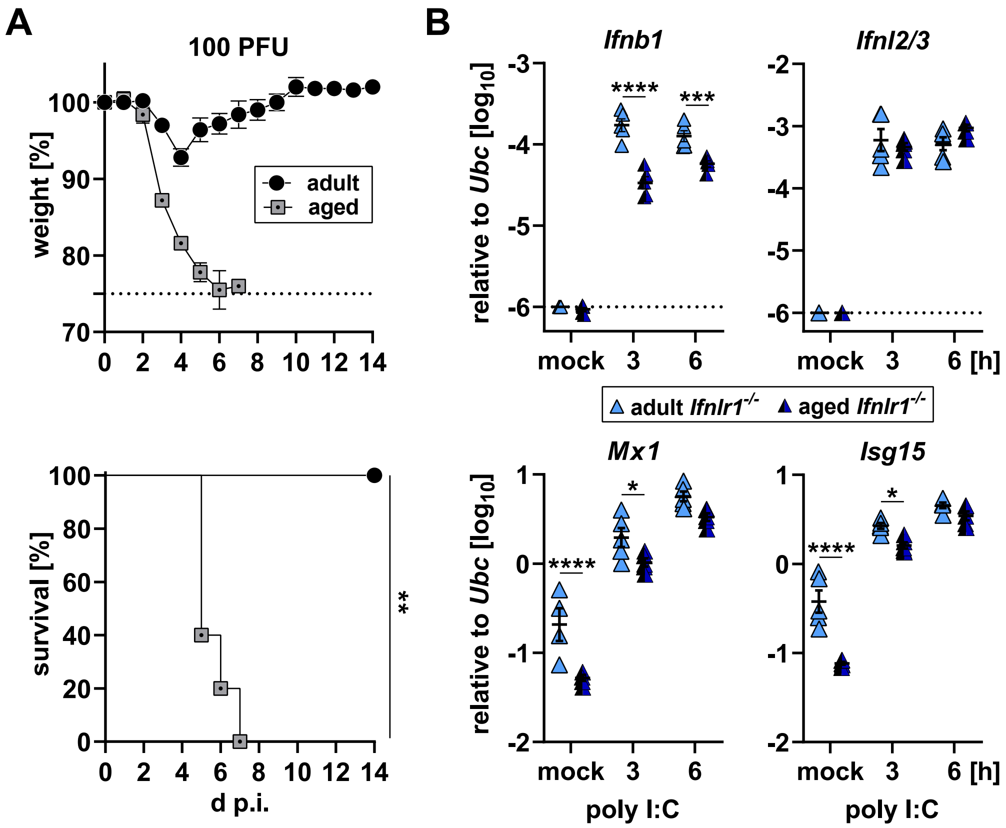
A
100 PFU
weight [%]
adult
aged
survival [%]
**
d p.i.
B
Ifnb1
Ifnl2/3
relative to Ubc [log10]
****
***
mock
3
6
6 [h]
adult Ifnlr1-/-
aged Ifnlr1-/-
Mx1
Isg15
*
****
poly I:C

Figure S3

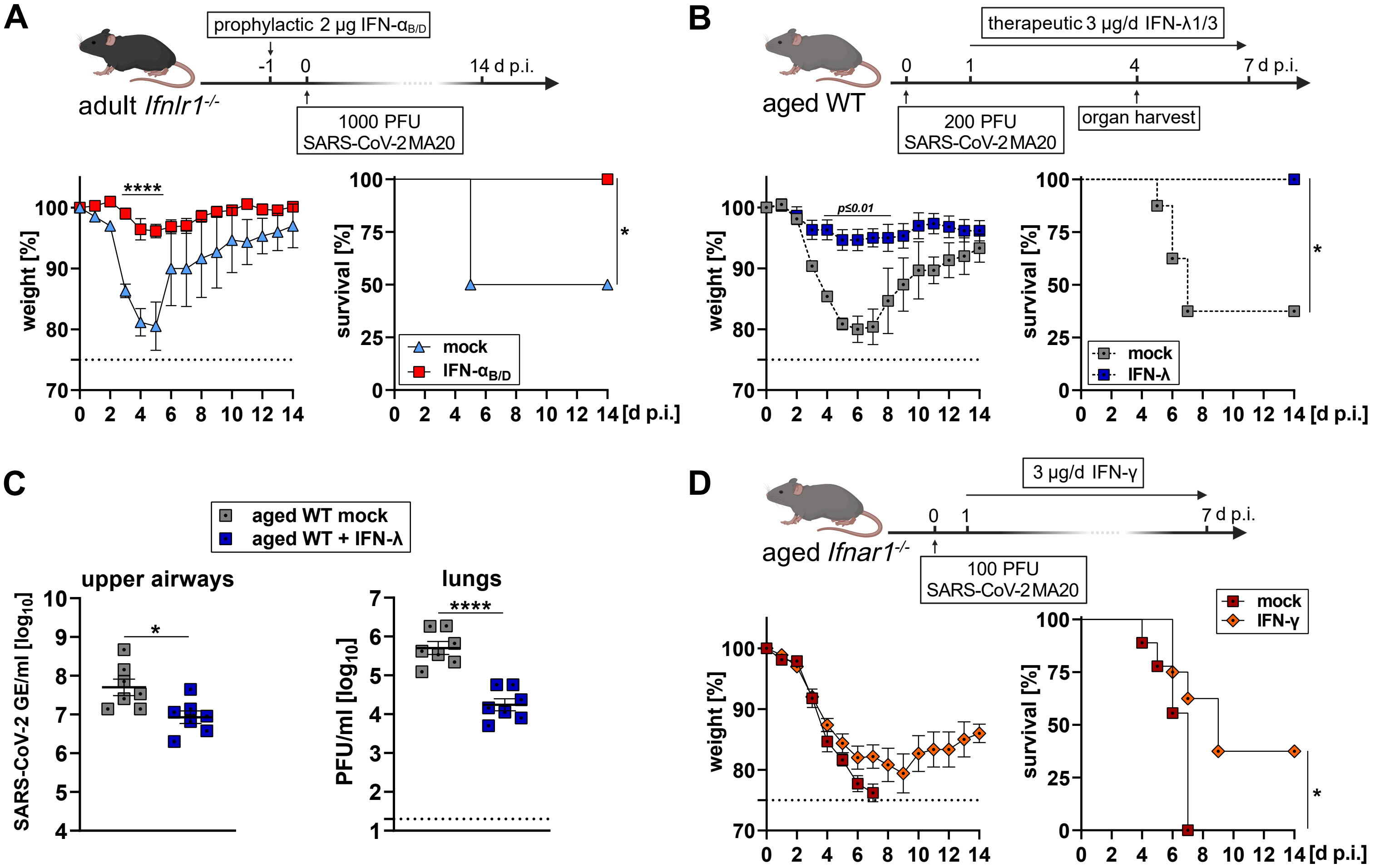
A
prophylactic 2 µg IFN-αB/D
-1
0
14 d p.i.
adult Ifnlr1-/-
1000 PFU
SARS-CoV-2 MA20
weight [%]
****
survival [%]
*
mock
IFN-αB/D
[d p.i.]
B
therapeutic 3 µg/d IFN-λ1/3
0
1
4
7 d p.i.
aged WT
200 PFU
SARS-CoV-2 MA20
organ harvest
p≤0.01
mock
IFN-λ
C
aged WT mock
aged WT + IFN-λ
upper airways
lungs
SARS-CoV-2 GE/ml [log10]
PFU/ml [log10]
D
3 µg/d IFN-γ
aged Ifnar1-/-
100 PFU
SARS-CoV-2 MA20
mock
IFN-γ

Figure S4

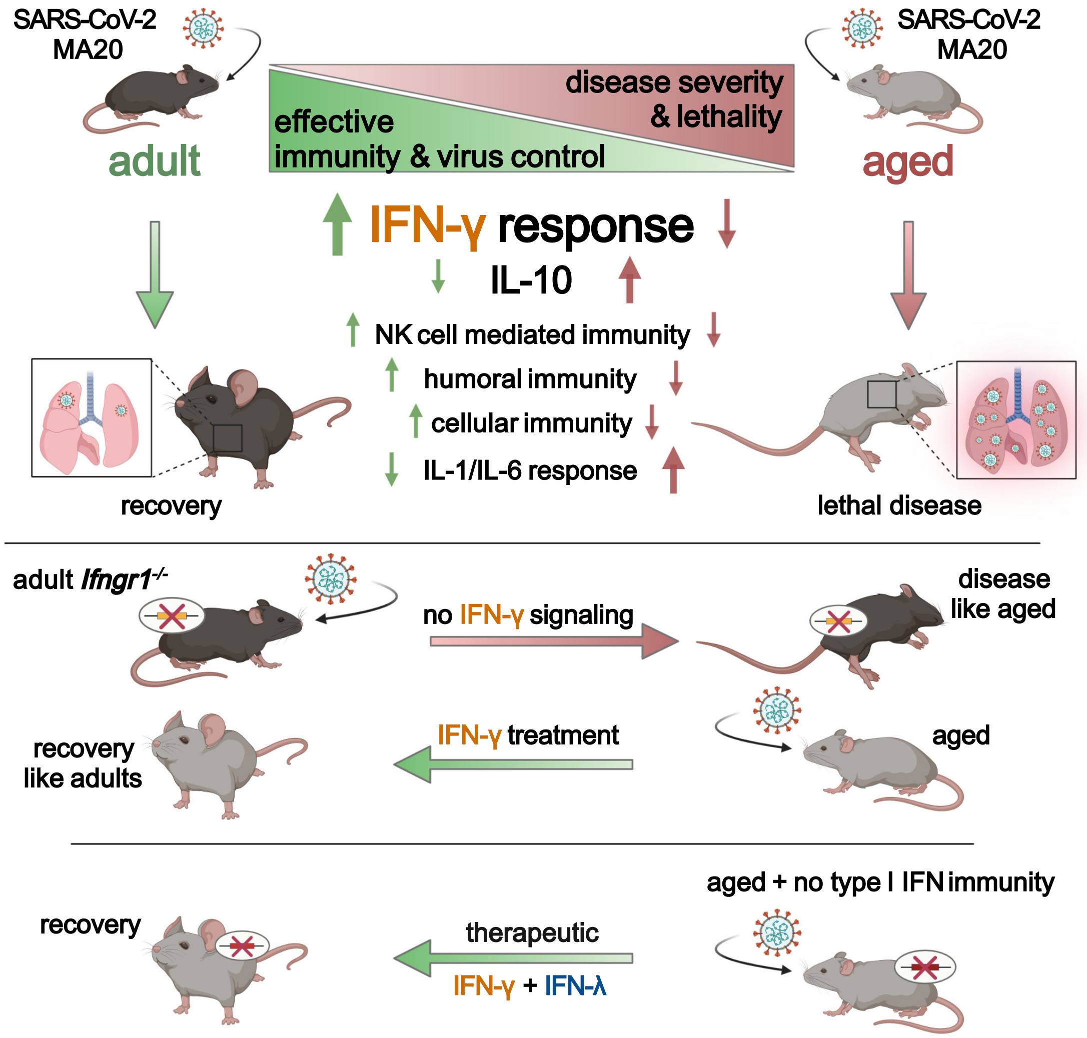
SARS-CoV-2
MA20
adult
effective
immunity & virus control
disease severity
& lethality
SARS-CoV-2
MA20
aged
IFN-γ response
IL-10
NK cell mediated immunity
humoral immunity
cellular immunity
IL-1/IL-6 response
recovery
lethal disease
adult Ifngr1-/-
no IFN-γ signaling
disease
like aged
recovery
like adults
IFN-γ treatment
aged
aged + no type I IFN immunity
recovery
therapeutic
IFN-γ + IFN-λ

Figure S5
